# Health and Climate at COP29: Advancing Integration and Bridging Research Gaps

**DOI:** 10.5334/aogh.4895

**Published:** 2025-09-25

**Authors:** Rupa R. Patel, Yevheniia Varyvoda, Hiba Baroud, Philip J. Landrigan, Aminata Kilungo

**Affiliations:** 1 Division of Infectious Diseases, Washington University in St. Louis School of Medicine, St. Louis, MO 63110, USA; 2 Friends in Village Development Bangladesh, Bangladesh; 3 The University of Arizona, Tucson, AZ 85721, USA; 4 Vanderbilt University, Nashville, TN 37235, USA; 5 Boston College, Chestnut Hill, MA 02467, USA; 6 Centre Scientifique de Monaco, MC 98000, Monaco

**Keywords:** COP, climate change, mitigation, adaptation, health, UNFCCC, RINGO

## Abstract

Climate and health science is a rapidly growing, interdisciplinary field and holds importance during intergovernmental climate adaptation and mitigation policy negotiations. The 29th session of the Conference of the Parties (COP) to the United Nations Framework Convention on Climate Change (UNFCCC) convened stakeholders for negotiations and scientific discourse. This viewpoint presents insights gained by the authors’ participation at COP29 focused on health and climate. The authors analyze the evolving integration of health into the UNFCCC process, dissect the contributions and the role of the Research and Independent Non‑Governmental Organizations (RINGOs), a constituency to achieve this endeavor, and identify the key gaps in public health research and the policy dialogue that persist in climate negotiations. In the context of bridging research with policy discourse, we define the role of and opportunities to engage meaningfully for young scientists in shaping climate and health within the UNFCCC framework, as well as advancing climate and health science.

## Introduction

Climate and health science is a rapidly growing, interdisciplinary field and holds importance during intergovernmental climate adaptation and mitigation policy negotiations. The 29th session of the Conference of the Parties (COP) to the United Nations Framework Convention on Climate Change (UNFCCC) convened stakeholders for negotiations and scientific discourse. This viewpoint presents an analysis of the evolving integration of health into the UNFCCC process, the role of the Research and Independent Non‑Governmental Organizations (RINGOs), a constituency to achieve this endeavor, key gaps in research, and opportunities for young scientists in shaping climate and health within the UNFCCC framework and progressing science.

## Integration of Health into the UNFCCC Process

The COP serves as the supreme body of the UNFCCC [1]. At present, there are 198 parties (197 countries and the European Union) that meet annually, unless otherwise determined, to review the progress made in implementing the Convention and any legal instruments adopted by the COP. During these meetings, decisions are made to promote the effective implementation of the Convention, including both institutional and administrative arrangements [1–3].

The intersection of climate change and health has gained increasing recognition within the UNFCCC negotiations. Initially overlooked in early climate discussions, health has progressively become a significant aspect of climate policy. The early years (1992–2007) saw minimal attention to health, with the UNFCCC (1992) and Kyoto Protocol (1997) focusing on greenhouse gas reductions rather than human health impacts [4, 5]. Reports from the World Health Organization (WHO) and Intergovernmental Panel on Climate Change (IPCC) in the 2000s highlighted climate‑related health risks such as vector‑borne diseases, heat‑related illnesses, and malnutrition [6, 7]. From 2008 to 2015, health integration gained traction, with the WHO launching the Climate Change and Health Program (2008) and COP16 (2010) recognizing health under adaptation policies [8, 9]. By 2015, the Paris Agreement explicitly acknowledged “the right to health,” urging countries to include health in Nationally Determined Contributions (NDCs) and National Adaptation Plans (NAPs) [10]. The momentum continued from 2016 to 2020, as the WHO launched the Special Initiative on Climate Change and Health (2017), COP meetings introduced Health Pavilions, and reports emphasized the health benefits of climate action and the need for climate‑resilient health systems [11, 12]. A key event in this trajectory was the first Global Conference on Air Pollution and Health, jointly organized in October 2018 in Geneva by the WHO, the World Meteorological Organization, UN Environment, and the Secretariat of the UNFCCC. This high‑level meeting brought together global, national, and local leaders in environmental health, climate change, and NCD control with the goal of formulating comprehensive strategies to reduce air pollution and curb NCDs while mitigating climate change and protecting the health of vulnerable populations.

Since 2021, health has become a central focus in climate negotiations. COP26 (2021) saw over 50 countries committing to climate‑resilient and low‑carbon health systems [13]. COP27 (2022) formally included health in the Sharm el‑Sheikh Implementation Plan, and the first‑ever Climate and Health Ministerial Meeting took place [14]. At COP28 (2023), “Health Day” was introduced for the first time, addressing climate‑induced heat‑related illnesses, infectious diseases, and mental health concerns [15]. Key themes in climate‑health discussions include climate adaptation for health systems, the health co‑benefits of climate mitigation, air pollution, food security, climate‑induced psychological stress, healthcare industry decarbonization and climate mitigation efforts, health financing within climate budgets, and the inclusion of public health within loss and damage funding [16]. At COP29, the Initiative on Human Development for Climate Resilience and the Multisectoral Action Pathways (MAP) Declaration for Resilient and Healthy Cities aimed to further integrate some aspects of health and the factors that drive health outcomes into climate resilience planning.

With COP30 a few months away, the 2025 Global Conference in Belem, Brasilia, on Climate and Health has set up the stage to continue pushing for health in the climate discussion. Part of the Belem objectives were to shape the Belem Health Action Plan (BHAP), enhance health‑climate government, advance ATACH commitments, and bridge evidence to action. The Belem Health Action draft is a significant progress as it anchors health as a central policy of climate health and will be adopted during COP30 [17].

There have been challenges in securing sufficient funding for health‑focused climate policies, but with the UNFCCC recently recognizing health as a fundamental component of climate action, ongoing, multistakeholder‑led efforts have been strengthened [15, 16].

With the current movement led by the WHO and several health sector advocates, future climate policies are expected to further prioritize and integrate health. Accelerated progress is imaginable but could be dampened by recent changes in country‑level financing and negotiation commitments, as well as funding losses for prominent organizations, such as the WHO [18]. There will be an important role for creative advocacy and financing strategies, even stronger and new multi‑stakeholder collaboration, coordination of organizational efforts, funding source diversification, and young advocate and scientist leadership to drive progress beyond the pace we had in the past.

Crucial next steps and the call to action include having scientists and policymakers expand the health priorities within NDCs, NAPs, and Loss and Damage Fund budgets, as well as help sustain the WHO’s core leadership role in these efforts.

## Role and Objectives of RINGO Climate Change and Health in the UNFCCC

The growing momentum to advance the climate change and health agenda within UNFCCC negotiations has also led to increased engagement from civil society, academia, and research groups, which play a crucial role in both advocacy and scientific contributions. The RINGO constituency was formed to represent officially admitted observer organizations of the UNFCCC negotiations process that are engaged in research, teaching, and other knowledge, practice, or theory‑based activities related to any aspect of climate change. RINGO is the second largest of the nine NGO constituencies recognized by the UNFCCC, comprising 25% of the 2,000 admitted NGOs. RINGOs promote effective research‑based outreach and capacity building on climate change for all stakeholders through seven thematic groups, including agriculture, Article 6, cryosphere, finance, public health, ocean, and technology [19, 20].

The RINGO Climate Change and Public Health Group (Health Group), established in 2023, actively participates in UNFCCC discussions. While still in its early stages, the group has expanded to over 50 members representing over 40 academic institutions and non‑profit organizations around the globe, working collectively toward three key objectives:

1. Generating evidence‑based knowledge on the health impacts of climate change to inform climate policy and negotiations.
2. Facilitating interdisciplinary collaboration among researchers, policymakers, and practitioners to strengthen the integration of health into climate dialogues.
3. Mentoring and training the next generation of scientists and stakeholders to actively participate in UNFCCC processes and contribute to climate‑health policymaking.

The RINGO Climate Change and Health Group has organized side events at COP meetings and other forums to convene diverse stakeholders, share research findings, and foster discussions on the health implications of climate change. Health Group member participation in UNFCCC negotiations remains a key mechanism for advancing climate‑health integration, ensuring that health considerations are adequately addressed in climate policy. The group is currently exploring ways to contribute to the IPCC by supporting the scientific assessment of climate and health risks, as well as generating mechanisms for experts to effectively disseminate their study findings to relevant local health ministries and country negotiators to inform NDCs, NAPs, negotiations, and other policy discussions.

The Health Group strives to grow their mentorship activities for emerging scientists and policymakers and support their active attendance and participation at COP and related local and international events. Providing experiences for young investigators to work, teach, and publish with colleagues and mentors from different disciplines within the field of climate and health (e.g., medicine, public health, economics, community advocacy, etc.) is critical in developing the next generation of leaders. To achieve this goal, synergizing efforts and resources with established university programs and networks is a priority. Cumulatively, these efforts aim to bridge the gap between science and policy, ensuring that health remains a central pillar of climate action within the UNFCCC framework.

## Gaps in the Current Public Health Research and Policy Dialogue at COP29

COP29 ingrained health as a non‑negotiable argument for climate action across people, place, and planet, prioritizing the urgent need to end reliance on fossil fuels and ensure people‑centered adaptation and resilience [21]. By attending and exploring climate‑health events at COP29, the authors distilled key messages from the global health community, clearly articulating priorities for governments, policymakers, and other sectors. These priorities include the importance of gender and equity considerations, empowering youth and Indigenous communities, investing in the health workforce, advancing research and surveillance in environmental and occupational health, innovating climate education for health professionals, promoting climate‑specific health literacy, advancing climate and health storytelling, decarbonizing the healthcare sector, accounting for the health costs of climate action and inaction, integrating nature‑based solutions for health, measuring the climate resilience of health systems, aligning food systems, nutrition and climate action, integrating health into NDCs, enhancing early warning systems, scaling up heat resilience, attracting investments in climate and health sectors, implementing actions on extreme heat, unlocking the potential of anticipatory actions, mitigating the mental health effects of climate change, and preventing climate amplified diseases and epidemics to strengthen climate governance.

Despite setting these priorities, significant gaps persist in integrating climate change and health into UNFCCC discussions, limiting effective policy action. Key challenges include the lack of dedicated climate‑health finance [1, 14], weak integration of health co‑benefits in mitigation strategies [1, 13], and insufficient disease surveillance systems to track climate‑driven infectious diseases [1, 22]. Additionally, mental health remains largely absent from climate policies, despite rising climate‑induced psychological distress [16]. Low‑ and middle‑income countries (LMICs) face underrepresentation in climate‑health research, leading to disparities in policy influence [23]. Furthermore, while progress has been made in Loss and Damage Fund negotiations, the $300 billion committed is insufficient, and health‑related losses remain insufficiently addressed [1, 24]. To bridge these gaps, COP29 was expected to emphasize stronger financing mechanisms for climate‑resilient health systems [13], expand global health research collaborations, and implement evidence‑based adaptation strategies that prioritize the most vulnerable populations. While COP29 focused on financing health, the Brasilia Conference represented a main shift toward mainstreaming health into the climate agenda, producing a concrete plan, the BHAP for COP30, and anchoring health as the central component of climate policy. Strengthening scientific advocacy, policymaker engagement, and interdisciplinary collaboration will be crucial to ensuring that climate action is also public health action as we look forward to COP30.

## Pathways for Meaningful Engagement in the UNFCCC Process: Opportunities for Young Scientists

Addressing global climate change policymaking requires the inclusion of scientists from multiple disciplines, including health scientists, in the policymaking process. One such approach to achieve this goal is through science for diplomacy, which refers to the use of scientific collaborations among nations to address common problems and build constructive international partnerships. Science diplomacy operates at the intersection of science, policy, and international relations, fostering a dialogue between scientists and policymakers to ensure that evidence‑based decisions are made on global issues, such as climate change. For science diplomacy to succeed in its mission and effectively support global climate change policymaking, the involvement of young scientists is essential for several reasons. First, their participation ensures a diversity of opinions, experiences, and innovative ideas, which are vital for tackling multifaceted global issues like climate change. Young scientists represent the future of science and policy, and their engagement today can help shape a more informed, resilient, and equitable future. Notably, opportunities like those offered by the IPCC have provided young scientists with platforms to influence policy.

Despite the added value and clear benefit of including scientists in global climate change policymaking, young scientists face significant challenges in their participation in science for diplomacy. One key issue lies in the conflicting demands of traditional academic career paths and the broader societal impacts of their work. Academic success is often measured by metrics such as published papers and secured research grants. These metrics, while important, fail to recognize contributions to science diplomacy or policymaking, creating a misalignment between career incentives and impactful work. Additionally, the time and effort required for meaningful engagement in science diplomacy often come at the expense of traditional academic and scientific career responsibilities. As a result, many young scientists face the difficult choice between advancing their careers within academia or pursuing impactful policy engagement. This dichotomy highlights the need for institutional recognition of science diplomacy as a valid and rewarding career path, ensuring that the contributions of scientists to policy are valued alongside traditional academic achievements.

To facilitate the inclusion of scientists in diplomacy and empower young scientists to contribute and participate in science‑informed policymaking, mechanisms must be developed to first recognize science diplomacy as a career path and then provide the tools for young scientists to succeed in this career. Below are examples of specific actions that must be taken to advance toward inclusive science diplomacy:

- Establishing dedicated training programs and career pathways for scientists interested in science diplomacy to help align their aspirations with opportunities for impactful work. Such programs should focus on skills like communication, negotiation, and interdisciplinary collaboration.
- Recognizing interdisciplinary collaboration and policy implication as a form of scientific advancement by encouraging and rewarding teamwork across disciplines between science and policymaking to foster a culture of mutual respect between scientists and policymakers. Policy work and interdisciplinary collaboration should be recognized as hard career development objectives and career promotion milestones.
- Developing mentorship programs, webinars, and workshops that bring together scientists and policymakers. These initiatives allow both groups to learn from each other’s perspectives, fostering an environment where evidence‑based decisions are prioritized.

Young scientists hold immense potential to shape climate policy through their unique perspectives and expertise. Youth and early career researchers from diverse regions and disciplines can fill the niche of climate change science and policy translators, communicators, and influencers. They possess the professional capacity and skills to understand complex information, avoid misinformation and disinformation, and frame it within the socio‑economic, cultural, linguistic, environmental, and historical contexts of specific populations or audiences [3]. By integrating context with effective and innovative tools such as mixed media, they can craft messages that resonate, are easily understood, and inspire actions.

A range of accessible, low‑barrier resources is available for young scientists to engage meaningfully in climate‑health policymaking. The presented examples of pathways are framed by the Action for Climate Empowerment (ACE) elements.

*Building foundational expertise (education and training):* Young scientists can build and develop professional competencies in the domain of climate change and health through open‑access resources.

- Online courses: Platforms such as UNCC:e‑Learn and the WHO Academy offer specialized courses and resources on climate change and health, climate action, negotiation strategies, and planning. Examples include WHO Country Support on Climate Change and Health, WHO The Health Argument for Climate Action, WHO Academy Climate Change and Health Toolkit, UNCC:e‑Learn: Human Health and Climate Change, UNCC:e‑Learn: Climate Change Negotiations and Health, Climate Resilient Water Safety Plans [25].
- Training and fellowships: Organizations such as the Global Consortium on Climate and Health Education (GCCHE), the Association of Schools of Public Health in the European Region (ASPHER), and the One Health Workforce Academies, among others, offer specialized fellowships and continuing professional development programs. These initiatives are designed to build capacity and expand professional networks, increasingly featuring remote or hybrid modalities, multilingual delivery, and free or low‑cost options. By reducing financial, logistical, and language barriers, they expand access for a global cohort of young scientists.

*Leveraging data for public engagement and communication (public access to information and awareness):* Access to climate and health data sources, combined with AI‑driven tools, enables young scientists to translate complex information into compelling, evidence‑based narratives.

- Data hubs: Open‑access platforms such as the WHO Global Health Observatory, the Climate and Health Outcomes Research Data Systems (CHORDS) provide evidence‑ and context‑based data and information. Young scientists can use these resources to craft targeted communication and outreach materials—policy briefs, podcasts, chatbots, and visual art—that illustrate climate‑health risks and advocate for action.

*Pathways for direct participation and visibility enhancement (public participation and international cooperation):* Young scientists can engage in the policymaking process through multiple entry points, from local to international levels.

- Digital policy networks: Social media platforms are increasingly used as communication channels in the course of international climate negotiations [26]. By following relevant event and initiative pages, for example, Youth4Climate, using official event hashtags, and engaging directly with policymakers, young scientists worldwide can actively contribute to and influence policy debates within the UNFCCC process.
- Grassroots and volunteer initiatives: Participating in social movements driven by and targeting young scientists, volunteering with UN and other agency programs, or contributing to citizen science projects, such as collecting local community data, mapping case studies, documenting coping strategies, or developing storytelling, offers practical ways to contribute to building the evidence base on the health impact of climate change. These activities not only strengthen knowledge at the climate‑health intersection and help counter climate change misinformation but also build a portfolio of impact that can open new career opportunities.

These and other available pathways equip young scientists with the knowledge, skills, resources, and career development opportunities needed to influence climate‑health policy, design and implement actionable solutions, and amplify youth leadership in addressing climate and health challenges.

Despite the advances of integrating health into the policy dialogues of the UNFCCC framework and climate negotiations, particular interdisciplinary research gaps and their applications exist. The global community has an obligation to provide meaningful opportunities for young scientists to engage in the UNFCCC process, which can accelerate the progress in addressing scientific gaps, centering health in climate dialogue, and generating the next generation of leadership in the fight against climate change.

## Disclaimer Statement

The Research and Independent Non‑Governmental Organizations (RINGO) constituency to the UN Framework Convention on Climate Change (UNFCCC) thematic groups provide a space for researchers to share knowledge, best practices, research ideas, and other resources on specific topics. While thematic group members identify as RINGOs, the views of thematic group coordinators and members are their own. The coordinator and members do not speak on behalf of the UNFCCC RINGO constituency. 
